# Supplementary material for: Genetic mechanisms for size- and light-dependent reproductive maturation in two maple species, Acer amoenum var. amoenum and Acer mono var. glabrum
Source: Ann Bot. 2026 Apr 24;138(2):596–610. doi: 10.1093/aob/mcag111 (PMC13454698; doi:10.1093/aob/mcag111)
Supplement: Supplementary file 1 [file mcag111_supplementary_data.zip › AOB_Revision2_history.docx]

**Article type:** Original article

**Title:** Genetic mechanisms for size- and light-dependent reproductive maturation in two maple species, *Acer amoenum* var. *amoenum* and *Acer mono* var. *glabrum*

**Authors:** Maki Suzuki^1*^, Yukine Fujioka^1^, and Yoko Hisamoto^2^

^1^ Graduate School of Frontier Sciences, The University of Tokyo

Room 568, Environmental Building, GSFS, University of Tokyo, 5-1-5 Kashiwanoha, Kashiwa, Chiba, 277-8563 Japan

^2^ Graduate School of Agricultural and Life Sciences, The University of Tokyo

The University of Tokyo Chichibu Forest, 1-1-49 Hinodamachi, Chichibu, Saitama, 368-0034 Japan

**Running title:** Genetic mechanisms of reproductive maturation in two maple species

**For correspondence. E-mail:* [suzukima@edu.k.u-tokyo.ac.jp](mailto:suzukima@edu.k.u-tokyo.ac.jp)

**Abstract**

- **Background and Aims:** Wild trees have a long prematuration period before flowering. Threshold tree size and light conditions are suspected to trigger the reproductive maturation, though genetic evidence is needed to confirm this. We investigated the genetic mechanisms underlying the onset of reproduction using two wild maple species, *Acer amoenum* var. *amoenum* (Aa) and *A. mono* var. *glabrum* (Am).
- **Methods:** We performed RNA-seq on leaves and buds sampled from the canopy tops of wild juvenile and mature trees to capture RNA expression during bud formation. We identified genes whose expression levels were highly correlated with *FLOWERING LOCUS T* (*FT*) as well as with tree size and/or light conditions; such genes were designated as differentially expressed genes (DEGs). Genes significantly correlated with light were further examined through a light manipulation experiment.
- **Key Results:** Most DEGs were correlated with both tree size and light conditions, demonstrating clear expression changes between saplings and adult trees. In Am, DEG expression patterns in adult trees further relied on tree size and light conditions, unlike Aa, the shade-adapted species. DEGs included mRNA of several flowering pathway transcription factors, including SQUAMOSA promoter-binding protein-like, FRIGIDA-like, and COP1, indicating their involvement in the lifetime reproductive schedule of *Acer* trees. Some of these genes responded to light manipulation, demonstrating light-dependent adjustments.
- **Conclusions:** Overall, our results reveal a synchronous shift in flowering-related gene expression across tree size, along with interspecific differences correlated with their different light requirements.

**Key words:** tree reproductive maturation, *Acer amoenum* var. *amoenum*, *A. mono* var. *glabrum*, *FLOWERING LOCUS T*, age-dependent pathway, light-dependent pathway, *SQUAMOSA promoter-binding protein-like*, *CO-FT* module, *FRIGIDA-likes*

INTRODUCTION

Trees undergo decades-long prematuration periods before the onset of flower production. The length and determinants of these periods are of great importance to horticulturists, pomologists, and ecologists, though our understanding of these remains incomplete. According to evolutionary theory, the duration of the prematuration period in wild plants is hypothesized to reflect a trade-off between the risk of dying before producing sufficient offspring and benefits associated with greater reproductive success at later life stages (e.g., Harper and White, 1974; Kozłowski, 1992). Accordingly, past and recent ecological studies have aimed to test these hypothetical tradeoffs by examining the lifetime reproductive onset of wild trees (e.g., Thomas, 1996; Suzuki et al., 2019; Journé et al., 2024). Meanwhile, the transition from vegetative to reproductive phase has long been a central issue in molecular plant biology. Multiple regulatory mechanisms, including those related to aging, vernalization, day length, hormone levels (e.g., gibberellin), and autonomous pathways, have been identified as jointly controlling the seasonal reproductive onset of annual plants (e.g., Pajoro et al., 2014; Kinoshita and Richter, 2020; Niu et al., 2024). However, these mechanisms have yet to be integrated into our understanding of the lifetime reproductive onset of trees, which operates over a decadal timescale.

Existing evidence suggests that both internal and external factors influence the onset of reproduction in trees. Observations from fruit trees and model species showing that cuttings tend to have shorter prematuration periods than seedlings imply the existence of age-related reproductive triggers. The *SQUAMOSA promoter-binding protein-like* (*SPL*) gene, a key component of the age-related flowering pathway in *Arabidopsis* (Pajoro et al., 2014) and a contributor to age-related leaf morphology in trees (Ostria-Gallardo et al., 2016), is a plausible candidate for an age-related flowering regulator. However, an experiment using *Populus* cuttings (Hsu et al., 2011) did not support *SPL*’s involvement in reproductive maturation. Instead, two paralogs of *FLOWERING LOCUS T* (i.e., *FT1* and *FT2*) showed increased expression as trees aged, while *MADS7*, a paralog of the flowering repressor *SHORT VEGETATIVE PHASE* (*SVP*), was found to be active mostly in juvenile trees (Hsu et al., 2011). Meanwhile, as wild trees grow larger, their surrounding environment also changes, and this external shift can promote flowering in some species (Day and Greenwood, 2011). For example, forest trees tend to flower at smaller sizes when their growth rate is high under favorable environmental conditions (Shibata and Tanaka, 2002). This observation implies the involvement of light- or temperature-dependent pathway genes in addition to age-related pathway genes. Furthermore, the availability of resources, including carbon and nitrogen, generally increases as trees grow larger, thereby enabling costly flowering (Miyazaki, 2013, Miyazaki et al., 2014). The complex interplay between growth stages and environmental conditions has long hindered efforts to elucidate the mechanisms regulating the onset of reproduction in trees.

In this study, we aimed to characterize overall changes in the activity of flowering-related genes in wild trees in relation to their size and size-related shifts in the light environment. To this end, we conducted correlation analyses to identify all genes whose expression levels were associated with those of *FLOWERING LOCUS T* (*FT*) and varied with tree size and/or light conditions. Two maple species were selected for analysis: *Acer amoenum* Carrière var. *amoenum* (Syn.: *A. palmatum* Thunb. subsp. *amoenum*) and *A. mono* var. *glabrum* H. Hara (Syn.: *Acer pictum* Thunb. subsp. *mono*). Hereafter we refer to these species as “*A. amoenum*” and “*A. mono*” for simplicity. Maples have broad economic importance in horticulture, landscaping, and forestry, making their flowering behavior of interest across sectors. Both focal species demonstrate that the mass of flowers related to tree size and light conditions (Uraguchi and Kubo, 2005). Moreover, they exhibit distinct life history strategies tied to their physiological traits: *A. amoenum* leaves are adapted to shaded conditions (Oguchi et al., 2006), whereas *A. mono* exhibits high plasticity across varying light conditions (Tanaka et al., 2008). Notably, *A. amoenum* produces many flowers even in shaded environments and can begin flowering before reaching the canopy (Uraguchi and Kubo, 2005), whereas *A. mono* initiates flowering only upon reaching the canopy (Suzuki et al., 2019). Taken together, these differences suggest species-specific genetic control mechanisms for flowering. Specifically, flowering gene expression is expected to be more light-dependent in *A. amoenum* and more size-dependent in *A. mono*. We tested this hypothesis by examining the correlation between flowering gene expression, tree size, and light conditions. Since tree size and light availability are strongly correlated in natural settings, we also conducted a light manipulation experiment to disentangle their individual effects. Through these approaches, we sought to better understand the genetic mechanisms that regulate reproductive onset in wild trees.

MATERIALS AND METHODS

***Study sites and sampling design***
Field sampling was conducted in the Tomakomai Experimental Forest of Hokkaido University, Japan (TOEF; 42º43´N, 141º36´E). TOEF is located near the Pacific Ocean coast and belongs to the cool temperate region. According to meteorological data from the nearest climate station, the annual mean air temperature at TOEF in 2021–2022 was 8.8°C (range: −17.4°C to 33.8°C), with the coldest period occurring in winter when a 20–30 cm snow layer covers the forest floor. Annual precipitation at the study site was 1,303 mm in 2021 and 1,438 mm in 2022, with frequent rainfall during the summer (Japan Meteorological Agency). The forest’s topsoil comprises of a thick layer of volcanic ash from a major eruption of nearby Mt. Tarumae in 1667. Deciduous hardwoods such as *Quercus crispula*, *Ostrya japonica*, and the target species (*A. amoenum* and *A. mono*) form the canopy, together with evergreen conifers (e.g., *Abies sachalinensis* and *Picea jezoensis*).

Sampling was conducted in two natural forest stands within TOEF. Both stands were located 80 m above sea level and 700 m apart. Although each forest was approximately 200 years old with a canopy height of about 30 m, their canopy structures had been disturbed by infrequent typhoons and, at the second site, by selective harvesting. The first, unharvested site (hereafter referred to as the “crane site”; compartment #204) was equipped with a 40 m canopy crane, which enabled safe leaf sampling and accurate canopy height measurements. The second site (compartment #208), hereafter referred to as the “tower site,” included scaffolds built to allow canopy access (see details below). Based on monitoring over 2019 to 2024, the sampling years (2021 and 2022) were average blooming years for both *A. amoenum* and *A. mono* (Y. Fujioka, personal observation).

***Sampling in the crane site***
Seven trees of each target species were selected at the crane site (Table 1). For each tree, height (H, m) and diameter at breast height (DBH, cm) were measured in August 2020. Tree heights were measured from a crane gondola using a tape measure or from the ground using a measuring pole. Branches with a basal diameter of approximately 1 cm were selected on each tree. Photon flux density (PFD) at the treetop was measured on sunny days in August 2022 using a LIGHT ANALYZER LA-105 (Nippon Medical and Chemical Instruments Co., Ltd., Osaka), which simultaneously records photosynthetic photon flux density (PPFD; 400–700 nm), PFD-R (600–700 nm), and PFD-FR (700–780 nm). Relative photon flux density (RPFD, %) at each treetop was calculated by dividing the PPFD value by that of a nearby open control measured with the MQ-100 (Apogee Instruments, Inc., North Logan). Finally, the red to far-red light ratio (R:FR) was calculated from PFD-R and PFD-FR values.

Leaves and buds were sampled weekly from July 12 to August 3, 2021. This period began before primordial development and ended in mid–bud formation. Microscopic observations of 2022 buds indicated that flower development began between August 4 and August 17. On each sampling date, three replicate sets, each consisting of one leaf and its axial bud (if present), were collected from the upper canopy of each sample tree. Samples were immediately immersed in RNAlater™ Stabilization Solution (Invitrogen) in microtubes, transported at low temperature, and stored in a deep freezer until RNA extraction. For RNA-seq, only one of the three replicates per week was analyzed to reduce costs; the remaining samples were archived for future studies.

***Light manipulation experiment in the tower site***
Under natural conditions, tree height, DBH, and light environment (RPFD and R:FR) are highly correlated. To estimate their individual effects, we conducted an experimental manipulation at the tower site.
Three canopy trees, each 9–13 m tall, were selected for each species (Table 1). Height and DBH were measured in May 2022. In June, TOEF staff erected steel scaffolds around each of the six trees. Twelve branches with a basal diameter of ~1 cm were selected from each tree, nine from the upper canopy and three from a nearby self-shaded area. Three sunny branches served as “bright” controls (Table 2). Another three sunny branches were wrapped in shade cloth that reduced light of all wavelengths by 50% (“shade” treatment). The remaining three sunny branches were wrapped in MegaCool™ film (MKV Advance Co., Ltd., Tokyo), which reduced radiation <400 nm and 700–950 nm by ~50%, thereby increasing the red to far-red light ratio (“high-R:FR” treatment). The self-shaded branches served as “dark” controls. Treatments were applied in late June 2022. Sampling occurred three times at biweekly intervals from July to August 2022. However, only samples collected on July 21 or 22 were analyzed for this study. One leaf and its axial bud were sampled from each branch and processed as in the crane site. Thus, for every treatment condition applied per tree, we obtained three replicates per sampling day. To reduce costs, only one replicate per treatment per tree was used in the RNA analysis (3 trees × 4 treatments = 12 samples). RPFD and R:FR values for each branch were measured on August 21 and September 3, 2022, as described above.

***RNA-seq library preparation and sequencing***
Total RNA from sampled leaves was extracted using the RNeasy Plant Mini Kit (QIAGEN). All procedures involved the addition of 10% PVP (polyvinylpyrrolidone, FUJIFILM Wako Pure Chemical Corp.) and otherwise followed the manufacturer’s instructions. RNA was extracted from 80 leaf samples (7 trees × 4 weeks × 2 species at the crane site and 3 trees × 4 treatments × 2 species at the tower site). Samples were then sent to Novogene Co., Ltd. (China) for total RNA transcriptome analysis. RNA quantity and quality were assessed using a NanoDrop spectrophotometer (NanoDrop Technologies, Santa Clara, CA, USA). Samples with RIN >6.3 were retained for sequencing. Library preparation was performed using the NEBNext Ultra RNA Library Prep Kit (New England Biolabs, MA, USA). Paired-end, strand-specific sequencing of 150 bp fragments was conducted on a NovaSeq6000 platform (Illumina Inc., San Diego, CA, USA) by Novogene Co., Ltd. (Beijing, China). After sequencing, the data have been deposited with links to BioProject accession number PRJDB37500 in the DDBJ BioProject database.

Bioinformatics analysis was conducted using OmicsBox version 3.5. First, the quality of raw reads was assessed with FastQC version 0.11.08. Next, adapter sequences were removed and low-quality reads were trimmed using Trimmomatic version 0.38. The quality trimming parameters used were as follows: target length = 40, strictness = 0.5, and minimum length = 36. STAR 2.7.8a, Samtools 1.12, and gffread 0.11.4 were used to map the trimmed reads to the maple reference genome sequence. To address the concern that differences in phylogenetic distance might affect the mapping of both species to the same reference genome, we used the sequence of a species more closely associated with both maple species from the publicly available reference genome sequences. That is, *A. mono* mapping used the reference genome of *A. truncatum* (PRJNA557096; Ma et al., 2020) from the same Platanoidea section, whereas *A. amoenum* mapping utilized the reference genome of *A. pseudosieboldianum* (PRJCA006356; Li et al., 2022) from the same Palmata section (Gao et al., 2020). HTSeq 0.9.0 or 2.0.5. was used for transcript quantification. Qlucore Omics Explorer version 3.8 or R with edgeR was used to ultimately normalize the count data employing the Trimmed Mean of M-values method.

National Center for Biotechnology Information (NCBI) Blast+ 2.13.0 (database search conducted in September 2023) was used to assign annotation. A blastp-fast search against the UniProtKB/Swiss-Prot database (swissprot v5) was performed, restricted to Viridiplantae (NCBI taxonomy ID:33090) entries. Furthermore, NCBI Blast+ 2.17.0 (database search conducted in January 2026) was used to perform a blastp-fast search against the non-redundant protein sequence database (nr v5), restricted to entries for the genus *Acer* (NCBI taxonomy ID:4022). Blast2GO, based on the Gene Ontology database, was used for GO annotations.

***Statistical analyses***

Three *FLOWERING LOCUS T* (*FT*) gene transcripts were detected in the *A. amoenum* contigs using the *A. pseudosieboldianum* genome as the reference, though one of them was very low representation. The remining two transcripts were identified as two *FT* homologs of *A. amoenum* (*Aa-FT1*, *Aa-FT2*). These two alignments were also detected from *A. mono* contigs using *A. pseudosieboldianum* genome as the reference and were considered as *FT*s of *A. mono* (*Am-FT1*, *Am-FT2*) (see Appendix S1 for details).

Crane site samples collected across different seasons were treated as biological replicates to reduce variation due to seasonal effects unrelated to tree size. Tower site samples from the “bright” condition (three replicates per tree) were also included in correlation analyses. For each species, Pearson correlation coefficients were calculated between the normalized read counts of *FT* and those of all the other transcripts. Transcripts significantly correlated with *FT* expression were further tested for correlation against four variables: tree height, DBH, RPFD, and R:FR. The significance threshold for these *t*-tests was set at *p* < 0.001 to minimize the risk of Type-I error. This strict threshold, however, increased the risk of Type-II errors. To address this, known transcription factors (TFs) involved in flowering pathways were retrieved from review literatures. These included those regulating plant age (Niu et al., 2024; Chitwood and Sinha, 2016), light signaling (Endo et al., 2016), vernalization (Niu et al., 2024), gibberellin signaling (Kinoshita and Richter, 2020), and the autonomous pathway (Mimida et al., 2013). For transcripts showing similarity to any of these TFs, additional Pearson correlations were performed between their normalized expression and *FT* expression, as well as with the four explanatory variables. These additional *t*-tests used a threshold of *p* < 0.01 and were conducted using *R* 4.4.2 (*R* Core Team, 2024). For simplicity, genes identified through either approach are referred to as differentially expressed genes (DEGs) at each significance level (DEG_.001_ and DEG_.01_) in the following sections.

For each species, DEG expression patterns were visualized using heatmaps and hierarchical clustering via the *heatmap3* function in *R*. Next, GO enrichment analysis was performed to identify GO terms significantly overrepresented among DEGs, using Fisher’s exact two-sided test. For each species, GO terms were ranked by ascending *p*-values and retained until the cumulative Type-I error rate reached 0.05. This analysis was automated using a custom Perl script and the associated Statistics library. Enriched GO terms are interpreted as representing functions that vary with *FT* expression and with tree size and/or light environment.

Responses of DEGs to light manipulation were evaluated using experimental data recorded at the tower site. Linear regression models were fit using the *lmer* function in *R*. In the model, the normalized read count of each DEG was set as response variable, the RPFD and R:FR values were used as explanatory variables. The random tree-level effects were also considered, but in cases the random effect was non-significant (i.e., the model reported “singular fits”), a standard linear model was used instead (i.e., *lm*). The significance of regression coefficients was tested via *t*-test (*p* < 0.05). All regression analyses were conducted in *R* version 4.4.2.

RESULTS

***General Trends of Developmental Changes***
Of the more than 28,000 annotated genes in each species, approximately one-fourth demonstrated no similarity to any known gene, and roughly half lacked the terms “flower” and “vegetative to reproductive” in their GONames (Supplementary Table S1). Additionally, approximately 200 transcripts were observed in fewer than five samples in both species. After removing these low-representation transcripts, correlation analyses were conducted on approximately 1,400 transcripts for each species. *Aa-FT1* did not correlate with almost any other transcript, while *Aa-FT2*, *Am-FT1*, and *Am-FT2* significantly correlated with many other transcripts (Supplementary Fig. S1). These results did not change with the reference genome used. Of the transcripts that were correlated with *FT1/2*, 54 *A. amoenum* transcripts from *A. pseudosieboldianum*-referenced data and 47 *A. mono* transcripts from *A. truncatum*-referenced data were correlated with at least one from H, DBH, RPFD, or R:FR (Fig. 1). These small fractions of transcripts were considered DEG_.001_ (Supplementary Table S2).

In both species, about half of DEG_.001_ were correlated with all of the four parameters (Fig. 1). However, the contribution of RPFD remains unclear because all these DEGs correlated with RPFD were also correlated with H and R:FR. All but one DEG_.001_ in *A. amoenum* were correlated with R:FR. All *FT1*-related DEG_.001_ in *A. mono* were correlated with H and R:FR, whereas all *FT2*-related DEG_.001_ were correlated with H and DBH.

The heatmaps drawn for *FT*s and DEG_.001_ primarily correspond with tree height (H) in both species. Saplings (H < 5 m) and larger trees demonstrated contrasting expression patterns (Figs. 2, 3). A cluster of DEGs_.001_ in *A. mono* that were downregulated in adults was further divided into two subclusters (Fig. 3). One subcluster demonstrated remarkable depression in the tallest trees (H > 17 m), whereas the other was upregulated in shaded trees (RPFD < 50% and R:FR < 1). Conversely, sub-clustering of *A. amoenum* DEGs_.001_ did not correspond to tree size or light conditions (Fig. 2).

*Aa-FT2*, *Am-FT1*, and *Am-FT2* themselves were significantly upregulated in tall trees and high R:FR conditions (Fig. 4). In both species, both *FT1* and *FT2* belonged to the same cluster that was upregulated in tall trees (high H) (Fig. 2, 3). Some flowering-related genes, including *HD16*, *RUP2* (*A. mono*), *ATX1* and *INVE* (*A. amoenum*), belonged to the same cluster with *FT*s, indicating the size-related activation of these flowering processes (See full descriptions of these genes in Table S2). Further, the age-related pathway gene *SPL3* appeared in the same cluster with *FT*s in *A. amoenum*. Other DEGs showed an opposite expression pattern to *FT*s and were downregulated in taller trees. Autonomous-pathway flowering repressors *FRIGIDA-LIKE* 3 (*FRL3*) and *4A* (*FRL4A*, *A. mono*) belonged to this cluster.

***GO Enrichment***
Among the DEGs of *A. amoenum* and *A. mono*, 117 and 96 GO terms, respectively, were significantly enriched (cumulative Type-I error rate of Fisher’s exact test < 0.05; Table 3). Many of these terms were related to the regulation of transcriptional processes and the development of reproductive organs and related functions. Terms associated with hormonal processes were also frequent. Terms related to light response were also commonly observed, especially in *A. amoenum*. Further, terms related to short-term flowering regulation, including circadian rhythm and vernalization, were significantly enriched. These functional terms tended to be expressed or repressed as *FT* increased and trees grew.

***Changes in Expression Levels in Flowering-Related Genes***
We identified 66 and 63 kinds of known flowering pathway genes and their paralogs in RNA-seq data of *A. amoenum* and *A. mono*, respectively (Supplementary Table S3). Further, 11 transcripts of *A. amoenum* and 12 transcrips of *A. mono* annotated with those gene names demonstrated significant correlation (*p* < 0.01) with both *FT* and tree size and/or light environment (DEG_.01_). Table 4 summarizes their response to changes in tree size and light environment. The DEG_.01_ of the same pathway group exhibited similar expression patterns irrespective of the tree species.

Among the DEGs_.01_ of the light-dependent pathway, *cryptochrome DASH* (*CRYD*), which participates in repairing UV-damaged DNA, increased expression in tall trees and in high light condition (Table 4). Conversely, other photoreceptor genes of *A. mono* and *CONSTANS-LIKE 4* (*COL4*) of both species were underexpressed as trees grew and received more light (note: the correlation between *COL4* and *Am-FT1* was marginally significant; *p* = 0.011). In contrast, the regulators of the *CO-FT* module, e.g. *COP1* (Fig. 5)*, PHYTOCHROME-DEPENDENT LATE-FLOWERING* (*PHL*) of *A. amoenum,* and *REPRESSOR OF UV-B PHOTOMORPHOGENESIS 2* (*RUP2*) of *A. mono* tended to be more highly expressed in large trees and in bright conditions.

Among autonomous pathway genes, *FRIGIDA*-like genes (*FRL4A* in *A. mono* and *FRL3*) and *Flowering time control protein FCA* in *A. amoenum* demonstrated a negative correlation with *FT* expression (Fig. 5, Table 4). *FLOWERING LOCUS C* (*FLC*), the target gene of *FRL*s and the key repressor of *FT*, exhibited significant negative correlations with *FT*2, size, and light environment in *A. mono*, while it showed no correlation with *FT*s or other parameters in *A. amoenum* (*p*> 0.05; Fig. 4).

The expression levels of *SQUAMOSA promoter-binding protein-like* family genes (*SPL*s) of the age-related pathway increased with tree size and were positively correlated with *FT* expression, except for *SPL12* (Table 4, Fig. 6). This is consistent with the known role of SPLs, which are promoted by aging-mediated reduction of miR156 and subsequently repress miR172, thereby promoting *FT* expression. The expression of two sugar-mediated pathway TFs, CTR1 and EDR1, decreased with tree size and/or increased light, whereas another *CTR1*-homolog showed a contrasting trend.

***Light Manipulation Experiment***
Among all DEG_0.001_ and DEG_0.01_, significant responses to RPFD and/or R:FR manipulation were observed only in 12 DEGs of *A. amoenum* and 11 DEGs of *A. mono*, based on regression coefficients (Table 5). Except for *CRYD*, which is involved in repairing UV-damaged DNA, no light-dependent pathway genes showed significant response to light manipulation. In contrast, other flowering pathway genes showed significant response to light manipulation. In *A. amoenum*, autonomous pathway genes changed to facilitate *FT* expression in shaded branches, where *FRL3* was downregulated and *FCA* was upregulated. In contrast, *FRL4A* in *A. mono* was upregulated in low-RPFD branches, being repressive of *FT* expression. Age-related pathway genes *SPL4/5* were significantly downregulated by high R:FR in both species. *GAI-ASSOCIATED FACTOR 1* (*GAF1*), a core mediator of gibberellin-dependent *FT* regulation (Kinoshita and Richter, 2020), was significantly downregulated under bright conditions in *A. mono*. Other genes in Table 5 were annotated as having functions other than flowering, or as ubiquitin-conjugating enzymes (UBCs) and MYB-like proteins, whose functions are unlimited to flowering.

DISCUSSIONS

Contrary to our hypothesis, flowering gene expressions relied on both size and light environments in the two *Acer* species. However, the two species demonstrated different light responses in multiple flowering pathways. These results indicate that multiple flowering pathways contribute to species-specific light use habits. Here, we investigated the overall patterns of all DEGs and changes in known transcription factors, focusing on their generality and diversity.

***Overall trends in flowering-related gene expression***
Many flowering-related genes changed their expression levels with tree size and light environment, consistent with previously identified size- and light-dependent flowering trends of both species (Uraguchi and Kubo, 2005). Overall, the DEGs were associated with tree size, drastically changing between saplings (H < 5 m) and large trees (H > 9 m). Further, some DEGs in *A. mono* were dependent on R:FR. This is consistent with previous knowledge that *A. mono* is more light demanding than *A. amoenum* (Tanaka et al., 2008) and exhibits highly plastic responses to changes in light environment (Oguchi et al., 2006). Our observations at TOEF indicated that the flowering intensity of *A. mono* depended on both tree height and light environment, specifically the relative canopy height in association with competing trees (Fujioka and Suzuki, unpubl. res.). The predominantly size-dependent and partly light-dependent DEG expression in *A. mono* may contribute to this double-standard flowering.

The heatmap for *A. amoenum* demonstrated a drastic change between saplings and large trees (H > 9 m); however, changes in the expression among large trees did not reflect tree size or light conditions. DEGs in this species may be more sensitive to changes in light under dark conditions, including an R:FR ratio of 0.5–1.0. This is consistent with the previous knowledge that *A. amoenum* is more shade tolerant (Oguchi et al., 2006, Tanaka 2008). At TOEF, we observed double-standard flowering, i.e., size- and light-dependent flowering, also for this species. However, unlike *A. mono*, this species began flowering well below the forest canopy (Fujioka and Suzuki, unpubl. res.). The lack of size- and light-dependent patterns after the adult stage may represent the shade-adaptive habits of *A. amoenum*.

***Gene Ontology***

Many DEGs in both species were associated with GO terms related to translational processes and hormonal regulation, suggesting the importance of these pathways in lifetime reproductive scheduling. A limitation of the present analysis is that the DEGs included genes acting downstream of *FT*; nonetheless, several were known upstream regulators. For example, the gibberellin-dependent pathway is a major flowering control mechanism (Mutasa-Göttgens and Hedden, 2009) and likely interacts with other pathways (Bolouri Moghaddam and Van den Ende, 2013; Endo et al., 2016; Blanco-Touriñán et al., 2020).

Interestingly, genes associated with annual flowering control also changed expression over decades. For example, genes involved in vernalization and circadian rhythm shifted in expression with tree size in both species. Many DEGs were also linked to GO terms associated with epigenetic regulation (Table S2), which is essential in perennial plants, where *FLC* must be iteratively activated and repressed (e.g., Nishio et al., 2020; Niu et al., 2024). In addition, light-responsive genes varied with tree size. These results suggest a layered regulatory system in which long-term developmental cues influence annual flowering regulators.

***Responses of known flowering control pathways***

Key regulators from multiple flowering pathways exhibited meaningful changes in expression. Overall, these changes were supportive of flowering. As the trees grew, age-related pathway genes (*SPL*s) were upregulated, while the *FT*-repressors of the autonomous-pathway were down regulated, and the light-dependent pathway genes engaged in the *CO-FT* module were upregulated. These changes are examined in the following sections.

1. Age-related pathway genes: *SQUAMOSA promoter-binding protein-like*s (*SPL*s)

In *Arabidopsis*, the expression of the transcription factor *SPL* increases as *miR156* production declines with age, leading to the induction of *miR172* and the subsequent promotion of *FT* transcription (Pajoro et al., 2014). In this study, several *SPL* homologs were identified as DEGs. Except for *SPL12*, their expression level generally demonstrated a significant positive correlation with *FT* and increased with H, DBH and R:FR (Fig. 6). In natural populations, tree age was correlated with DBH, which further correlates with tree height in our study site (*r* = 0.98 for *A. amoenum* and *r* = 0.90 for *A. mono*). Therefore, height- and DBH-related expression changes in *SPL*s somehow reflect their age dependent changes. The miR156–SPL module has also been linked to the shade avoidance syndrome (Xie et al., 2017); however, most DEG *SPL*s showed no significant response in the light manipulation experiment. These *SPL* homologs are thus likely regulated by aging rather than light. *SPL5* in *A. amoenum* and *SPL4* in *A. mono* were significantly downregulated with an increase in experimental R:FR; however, these responses were contrary to previous knowledge on *Arabidopsis SPL*s that increase under dark conditions (Xie et al., 2017). Recently, gibberellin-dependent control of *SPL*s has been reported (Song et al., 2020), which may have contributed to our inconsistent results. Overall, *SPL* expression patterns were primarily aging-dependent, and opposing patterns by light and gibberellin were minor even if they existed (Table 3). The size- or age-dependent upregulation of *SPL*s may help in age-related flowering onset in the target species.

2. Autonomous pathway genes

Autonomous pathway genes such as *Flowering time control protein FCA, FRIGIDA-LIKE 3* (*FRL3*), *and FRIGIDA-LIKE 4A* (*FRL4A*) also exhibited significant changes in expression according to tree size and light environment. As trees grew, *FRL3/4A* were downregulated; this pattern was consistent with upregulation of *FT* during maturation. In *A. mono*, this pattern was shared by their target gene *FLC*. In this species, the expression level of *SHORT VEGETATIVE PHESE* (*SVP*), which is another key repressor of both *FT* and *SUPPRESSOR OF CONSTANS OVEREXPRESSION 1* (*SOC1*), also declined as trees grew. These changes may influence the lifetime reproductive timing of *A. mono*. Conversely, *FLC* and *SVP* expressions of *A. amoenum* demonstrated no significant correlation with *FT* expression, size, and light environments. In this species, lifetime flowering may be more regulated by other pathways than *FLC-*dependent ones.

The size- or light-dependence of the above changes may vary among the genes. The *SVP* expression is likely size-dependent, because an age-related change has been reported for its *Populus* homolog *MADS7* (Hsu et al., 2011). The lack of significant light response for *SVP* in our experiment further supports its size-dependent regulation. In contrast, *FCA*, *FRL3*, and *FRL4A* responded significantly to the light manipulation experiment, thereby supporting their sensitivity to the light environment of branches. A promoter analysis on soybean FRIGIDA-like proteins revealed that almost all FRLs were rich in light-responsive *cis*-acting elements (Yu et al., 2025). FCA is involved in temperature perception, and it changes its mRNA and protein levels in response to temperature (Jung et al., 2012). It likely responded to temperature changes based on light conditions. Thus, changes in FRLs and FCA were likely light-dependent, although size-dependent changes remain possible (e.g. hormonal control: (Yu et al., 2025).

Noteworthily, *FCA* and *FRL3* in *A. amoenum* were up- and down-regulated, respectively, on the shaded branches, supporting *FT* expression. Our observation in TOEF indicated that *A. amoenum* trees that compete with neighbors flower on shaded branches while reserving their topmost branches for elongation (Fujioka and Suzuki, unpubl. res.). This spatial allocation strategy could be realized by the light-dependent changes in *FCA* and *FRL3* expressions. Such a trend was not observed in *A. mono* trees, which typically flower after reaching the forest canopy. In this species, *FRL4A* was significantly downregulated in unshaded branches, promoting *FT* expression there. Based on these findings, we hypothesize the involvement of some autonomous pathway genes in the light-dependent adjustment of the flowering position within a tree canopy.

3. Light-dependent pathway genes

Most DEGs in both species were correlated with light conditions, specifically R:FR. These patterns suggest coordinated changes in light-regulated genes in response to size. Some of these size-dependent changes are unlikely to be linked to reproductive maturation. For example, the mRNA of phototropins 1 and 1B, which are receptors of weak blue light (Huber et al., 2021), declined under strong light. This reflects reduced demand for phototropins and a photoinhibition avoidance response (Miao et al., 2022). Further, the mRNA of cryptochrome-DASH, a potential photoreceptor and photolyase (Kiontke et al., 2020), increased under strong light that causes UV damage. Negative correlations of these mRNAs with *FT* are probably caused by pseudocorrelation.

However, some DEGs were involved in the *CO-FT* module of the light-dependent pathway, including *BBX31*, *COP1*, *PHL*, and *RUP2*. Size-dependent increase of these genes indicates coordinated upregulation of the light-dependent pathway. B-box domain protein 31 (BBX31) directly binds to CONSTANS (CO) protein and regulates FT production at dawn under long-day conditions (Takagi et al., 2023). *PHL* promotes *CO* and *FT* under weak light (Endo et al., 2016), supporting shade-adapted *A. amoenum* to flower. RUP2 protein is an essential flowering repressor under short-day conditions through its direct interaction with CO and suppression of *FT* transcription (Arongaus et al., 2018). These genes demonstrated no significant responses to light manipulation; thus, their size-dependent upregulation was more likely a life-cycle event than a response to the improved light conditions.

Unlike other light-dependent pathway DEGs, COP1 relates to *FT* expression in various ways. COP1 is a hub transcription factor in shade avoidance (Huber et al., 2021), functioning with SPA1 to regulate the circadian rhythm of the *CO-FT* module (Endo et al., 2016). COP1 also interacts with CONSTANS-like proteins (Ordoñez-Herrera et al., 2018). These functions may mediate the size-related upregulation of *FT* via COP1-related pathways. Furthermore, *COP1* is also known to degrade the protein DELLA, which is known to inhibit CO (Blanco-Touriñán et al., 2020), and may potentially activate the *CO-FT* module. Thus, the size-dependent COP1 upregulation may promote reproductive maturation in *Acer* via multiple mechanisms.

In the light-dependent pathway, not all DEGs were upregulated as trees grew. For instance, *COL4*, which aligned more closely with *COL5* of *Acer yangbiense* (Table S2), and *NF-YC9* (*Nuclear transcription factor Y subunit C-9*) were both significantly downregulated as trees grew. These genes promote flowering by driving the *CO-FT* module (Hassidim et al., 2009; Siriwardana et al., 2016); thus, their size-dependent decline contradicts our expectations. Further, many genes, including *CO*, exhibited no size-dependent changes in this study. These inconsistencies emphasize the complexity of the overall churning of the light-dependent pathway, warranting further investigation.

Lastly, some DEGs, including the above, were associated with daily and annual flowering regulation. Genes involved in vernalization (e.g., *JOINTLESS*, *RUP2*) and circadian rhythm (e.g., *BBX31*, *CIA2*, *COP1*, *LWD1*, *INVB*) exhibited expression changes associated with tree size. These results indicate a hierarchical regulatory system in which long-term developmental cues modulate annual flowering regulators.

**Conclusions**

This study revealed the responses of flowering-related genes from age-related, light-dependent, and autonomous pathways to tree size and light environment in the target species. Several of the changes were common to both target species; however, some interspecies differences were observed, particularly in their responses to light conditions. These patterns may provide a clue to the gene-level mechanisms that regulate the lifetime reproductive schedule of tree species. *Acer*, one of the most studied genera in plant reproductive ecology (e.g., de Jong, 1976), remains a promising model for this inquiry.

The present results have several limitations. Because we prioritized minimizing the risk of Type-I errors, many potentially important genes were excluded from analysis. We also failed to detect key factors whose expression levels did not correlate directly with *FT* expression. Further, many non-DEG homologs presented for known TFs, as in Table S3, are probably due to the very strict significance level and the uncertainty inherent to the short-read RNA-seq. Despite these limitations, our results provide an initial suggestion of where to apply more promising but expensive technologies. Further research is needed to clarify the mechanisms underlying the lifetime reproductive schedule of trees.

**Supplementary data**

Supplementary data are available at *Annals of Botany* online and consist of the following.

Appendix S1: Details on the *FLOWERING LOCUS T* detection process from the RNA-seq data. Table S1: Summary of the data filtering process. Table S2. The list of genes that were identified as DEGs in one or both target species. Annotations are based on Uniprot KB. Table S3: Correlation test results (*p* < 0.01) for transcripts annotated as major transcription factors of known flowering pathways. Figure S1: Venn diagram showing transcripts whose expression levels were correlated with *FT1/2* at a significance level of *p* < 0.001.

**Funding**

This work was supported by Japan Society for the Promotion of Science [KAKENHI Grant No. 21K19135] and Ministry of Education, Culture, Sports, Science and Technology-Japan [Joint usage / Education Center Program Grant No. 2023_06_D and 2024_07_D].

**Acknowledgements**

We thank Atsushi Okuda and all the staff at TOEF for their continuous cooperation and professional support throughout this study. Laboratory experiments were conducted using the laboratory of Prof. Kazuhide Nara. We are also grateful to Dr. Yuko Miyazaki for her helpful comments on this study, and to Mr. Rei Ito for his support on field sampling.

**Author contributions**

M.S. and Y.H. designed the research and acquired funding. Y.F. conducted most of the field survey, with support from M.S. and Y.H. Y.F. extracted RNA under the supervision of Y.H. M.S. and Y.H. performed the data analyses. M.S. interpreted the results in the discussion with Y.H. and Y.F. M.S. wrote the initial draft of the manuscript, which Y.H. and Y.F. thoroughly reviewed and edited. All authors approved the final manuscript.

**Conflicts of interests**

The authors report that there are no competing interests to be declared.

**Data availability**

All data contained in this article are available in the accompanying supplementary material.

**Literature cited**

**Arongaus A, Chen S, Pireyre M, Glöckner N, Galvao V, Albert A, Winkler J, Fankhauser C, Harter K, Ulm R. 2018.** Arabidopsis RUP2 represses UVR8-mediated flowering in noninductive photoperiods. *Genes & Development* **32**: 1332-1343.

**Blanco-Touriñán N, Legris M, Minguet E, Costigliolo-Rojas C, Nohales M, Iniesto E, García-León M, Pacin M, Heucken N, Bloemeir T, et al. 2020.** COP1 destabilizes DELLA proteins in *Arabidopsis*. *Proceedings of the National Academy of Sciences of the United States of America* **117**(24): 13792-13799.

**Bolouri Moghaddam M, Van den Ende W. 2013.** Sweet immunity in the plant circadian regulatory network. *Journal of Experimental Botany* **64**(6): 1439-1449.

**Day ME, Greenwood MS 2011.** Regulation of ontogeny in temperate conifers. In: Meinzer FC, Lachenbruch B, Dawson TE eds. *Size- and age-related changes in tree structure and function*. Dordrecht: Springer Netherlands, 91-119.

**de Jong PC. 1976.** *Flowering and Sex Expression in Acer L.: a Biosystematic Study.* Ph.D., Wageningen University and Research Netherlands.

**Dutta A, Choudhary P, Caruana J, Raina R. 2017.** JMJ27, an *Arabidopsis* H3K9 histone demethylase, modulates defense against *Pseudomonas syringae* and flowering time. *The Plant Journal* **91**(6): 1015-1028.

**Endo M, Araki T, Nagatani A. 2016.** Tissue-specific regulation of flowering by photoreceptors. *Cellular and Molecular Life Sciences* **73**(4): 829-839.

**Gadgil M, Bossert WH. 1970.** Life historical consequences of natural selection. *The American Naturalist* **104**(935): 1-24.

**Gao J, Liao P-C, Huang B-H, Yu T, Zhang Y-Y, Li J-Q. 2020.** Historical biogeography of *Acer* L. (Sapindaceae): genetic evidence for Out-of-Asia hypothesis with multiple dispersals to North America and Europe. *Scientific Reports* **10**(1): 21178.

**Harper JL, White J. 1974.** The demography of plants. *Annual Review of Ecology and Systematics* **5**: 419-463.

**Hsu C-Y, Adams JP, Kim H, No K, Ma C, Strauss SH, Drnevich J, Vandervelde L, Ellis JD, Rice BM, et al. 2011.** *FLOWERING LOCUS T* duplication coordinates reproductive and vegetative growth in perennial poplar. *Proceedings of the National Academy of Sciences* **108**(26): 10756-10761.

**Huber M, Nieuwendijk NM, Pantazopoulou CK, Pierik R. 2021.** Light signaling shapes plant–plant interactions in dense canopies. *Plant, Cell & Environment* **44**(4): 1014-1029.

**Japan Meteorological Agency. 2025. Serch for past weather data** [WWW document, In Japanese] URL <https://www.data.jma.go.jp/stats/etrn/index.php?prec_no=21&block_no=47424&year=2021&month=&day=&view=p1s>

**Jung J-H, Seo PJ, Ahn JH, Park C-M. 2012.** Arabidopsis RNA-binding Protein FCA Regulates MicroRNA172 Processing in Thermosensory Flowering. *Journal of Biological Chemistry* **287:** 16007-16016.

**Journé V, Bogdziewicz M, Courbaud B, Kunstler G, Qiu T, Acuña M, Ascoli D, Bergeron Y, Berveiller D, Boivin T, et al. 2024.** The relationship between maturation size and maximum tree size from tropical to boreal climates. *Ecology Letters* **27** (9).

**Kinoshita A, Richter R. 2020.** Genetic and molecular basis of floral induction in *Arabidopsis thaliana*. *Journal of Experimental Botany* **71**(9): 2490-2504.

**Kiontke S, Göbel T, Brych A, Batschauer A. 2020.** DASH-type cryptochromes – solved and open questions. *Biological Chemistry* **401**: 1487-1493.

**Kozłowski J. 1992.** Optimal allocation of resources to growth and reproduction: implications for age and size at maturity. *Trends in ecology & evolution* **7**(1): 15-19.

**Ma Q, Sun T, Li S, Wen J, Zhu L, Yin T, Yan K, Xu X, Li S, Mao J, et al. 2020.** The *Acer truncatum* genome provides insights into nervonic acid biosynthesis. *The Plant Journal* **104**(3): 662-678.

**Mimida N, Komori S, Suzuki A, Wada M. 2013.** Functions of the apple *TFL1/FT* orthologs in phase transition. *Scientia Horticulturae* **156**: 106-112.

**Miao L, Zhao J, Yang G, Xu P, Cao X, Du S, Xu F, Jiang L, Zhang S, Wei X, et al. 2022.** *Arabidopsis* cryptochrome 1 undergoes COP1 and LRBs-dependent degradation in response to high blue light. *New Phytologist* **234**(4): 1347-1362.

**Miyazaki Y. 2013.** Dynamics of internal carbon resources during masting behavior in trees. *Ecological Research* **28**(2): 143-150.

**Miyazaki Y, Maruyama Y, Chiba Y, Kobayashi M, Joseph B, Shimizu K, Mochida K, Hiura T, Kon H, Satake A. 2014.** Nitrogen as a key regulator of flowering in *Fagus crenata*: understanding the physiological mechanism of masting by gene expression analysis. *Ecology Letters*, 17: 1299-1309.

**Mutasa-Göttgens E, Hedden P. 2009.** Gibberellin as a factor in floral regulatory networks. *Journal of Experimental Botany* **60**(7): 1979-1989.

**Nishio H, Buzas DM, Nagano AJ, Iwayama K, Ushio M, Kudoh H. 2020.** Repressive chromatin modification underpins the long-term expression trend of a perennial flowering gene in nature. *Nature Communications* **11**(1): 2065.

**Niu F, Rehmani M, Yan J. 2024.** Multilayered regulation and implication of flowering time in plants. *Plant Physiology and Biochemistry* **213**.

**Oguchi R, Hikosaka K, Hiura T, Hirose T. 2006.** Leaf anatomy and light acclimation in woody seedlings after gap formation in a cool-temperate deciduous forest. *Oecologia* **149**(4): 571-582.

**Ordoñez-Herrera N, Trimborn L, Menje M, Henschel M, Robers L, Kaufholdt D, Hänsch R, Adrian J, Ponnu J, Hoecker U. 2018.** The transcription factor COL12 is a substrate of the COP1/SPA E3 ligase and regulates flowering time and plant architecture. *Plant Physiology* **176**(2): 1327-1340.

**Ostria-Gallardo E, Ranjan A, Chitwood DH, Kumar R, Townsley BT, Ichihashi Y, Corcuera LJ, Sinha NR. 2016.** Transcriptomic analysis suggests a key role for *SQUAMOSA PROMOTERBINDINGPROTEINLIKE*, *NAC* and *YUCCA* genes in the heteroblastic development of the temperate rainforest tree *Gevuina avellana* (Proteaceae). *New Phytologist* **210**(2): 694-708.

**Pajoro A, Biewers S, Dougali E, Valentim F, Mendes M, Porri A, Coupland G, Van de Peer Y, van Dijk A, Colombo L, et al. 2014.** The (r)evolution of gene regulatory networks controlling *Arabidopsis* plant reproduction: a two-decade history. *Journal of Experimental Botany* **65**(17): 4731-4745.

**R Core Team, 2024**. R: A Language and Environment for Statistical Computing. R Foundation for Statistical Computing, Vienna, Austria. <https://www.R-project.org/>

**Shibata M, Tanaka H 2002.** Reproductive traits of trees of OFR. In: Nakashizuka T, Matsumoto Y eds. *Diversity and Interaction in a temperate forest community Ogawa Forest Reserve of Japan*. Tokyo: Springer-Verlag, 95-108.

**Song M, Wang R, Zhou F, Wang R, Zhang S, Li D, Song J, Yang S, Yang Y. 2020.** SPLs-mediated flowering regulation and hormone biosynthesis and signaling accompany juvenile-adult phase transition in *Pyrus*. *Scientia Horticulturae* **272**: 109584.

**Suzuki M, Umeki K, Orman O, Shibata M, Tanaka H, Iida S, Nakashizuka T, Masaki T. 2019.** When and why do trees begin to decrease their resource allocation to apical growth? The importance of the reproductive onset. *Oecologia* **191**(1): 39-49.

**Tanaka H, Shibata M, Masaki T, Iida S, Niiyama K, Abe S, Kominami Y, Nakashizuka T. 2008.** Comparative demography of three coexisting *Acer* species in gaps and under closed canopy. *Journal of Vegetation Science* **19**(1): 127-138.

**Thomas S. 1996.** Relative size at onset of maturity in rain forest trees: A comparative analysis of 37 Malaysian species. *Oikos* **76**(1): 145-154.

**Uraguchi A, Kubo T. 2005.** Responses to light in individual growth and reproduction of two *Acer* species and the long-term consequences. *Ecological Research* **20**(2): 177-187.

**Xie Y, Liu Y, Wang H, Ma X, Wang B, Wu G, Wang H. 2017.** Phytochrome-interacting factors directly suppress MIR156 expression to enhance shade-avoidance syndrome in *Arabidopsis*. *Nature communications* **8**(1): 348.

**Yu S, Wang Y, Ren W, Fang Y, Wang L, Zhang Y, Song C, Luo X. 2025.** Comprehensive genome-wide analysis of the GmFRIGIDA gene family in soybean: identification, characterization, and expression dynamics. *Frontiers in Plant Science* **16** - 2025.

Table 1. Sample tree size and light environment.

| Species | Sampling Site | Height (m) | DBH (cm) | RPFD (%) | R:FR ratio | Flowering rate (%)^*^ | |
| --- | --- | --- | --- | --- | --- | --- | --- |
|  |  |  |  |  |  | *t* = 0 | *t* = 1 |
| *A. amoenum* | Crane site | 20.0 | 41.2 | 100 | 1.215 | 90 | 100 |
|  |  | 18.3 | 33.5 | 100 | 1.340 | 80 | 95 |
|  |  | 17.6 | 28.7 | 98 | 1.340 | 80 | 30 |
|  |  | 14.6 | 28.8 | 79 | 1.273 | 90 | 100 |
|  |  | 12.2 | 19.6 | 28 | 1.139 | 30 | 90 |
|  |  | 4.4 | 3.3 | 38 | 0.526 | 0 | 0 |
|  |  | 3.4 | 3.1 | 23 | 0.526 | 0 | 0 |
|  | Tower site | 11.4 | 15.0 | 100 | 1.216 | 95 | 5 |
|  |  | 10.5 | 15.4 | 100 | 1.166 | 70 | 5 |
|  |  | 9.2 | 14.4 | 100 | 1.208 | 95 | 10 |
| *A. mono* | Crane site | 17.8 | 27.2 | 100 | 1.318 | 60 | 80 |
|  |  | 17.4 | 47.3 | 100 | 1.261 | 60 | 60 |
|  |  | 16.5 | 44.6 | 86 | 1.229 | 60 | 90 |
|  |  | 11.3 | 19.0 | 21 | 0.985 | 30 | 60 |
|  |  | 10.5 | 10.8 | 32 | 0.908 | 0 | 30 |
|  |  | 4.1 | 2.7 | 9 | 0.526 | 0 | 0 |
|  |  | 4.6 | 2.3 | 35 | 0.526 | 0 | 0 |
|  | Tower site | 12.6 | 19.0 | 100 | 1.259 | 10 | 10 |
|  |  | 12.3 | 22.4 | 100 | 1.303 | 70 | 20 |
|  |  | 12.0 | 19.4 | 100 | 1.271 | 20 | 10 |

* Percentage of flowering current-year shoots in the year of RNA sampling (*t* = 0) and in the next sampling year (*t* = 1).

Table 2. Light conditions of experimental branch samples.

| Species | Treatment | RPFD(%) | | | R:FR | | |
| --- | --- | --- | --- | --- | --- | --- | --- |
|  |  | Mean | (Min | - Max) | Mean | (Min | - Max) |
| *A. amoenum* | Bright | 100.0 | (100.0 | - 100.0) | 1.197 | (1.166 | - 1.216) |
|  | Shaded | 45.7 | (21.0 | - 65.0) | 1.104 | (0.913 | - 1.229) |
|  | High-R:FR | 44.3 | (33.0 | - 57.2) | 1.429 | (1.221 | - 1.543) |
|  | Dark | 10.3 | (5.0 | - 21.1) | 0.451 | (0.351 | - 0.594) |
| *A. mono* | Bright | 100.0 | (100.0 | - 100.0) | 1.278 | (1.259 | - 1.303) |
|  | Shaded | 56.9 | (50.9 | - 60.8) | 1.262 | (1.231 | - 1.298) |
|  | High-R:FR | 63.8 | (57.2 | - 68.0) | 1.590 | (1.510 | - 1.703) |
|  | Dark | 25.6 | (21.1 | - 28.0) | 0.809 | (0.536 | - 1.116) |

Table 3. GO terms in which identified DEGs were enriched with significantly high probability.

| Functional groups of the terms | Examples | Number of enriched GO terms | |
| --- | --- | --- | --- |
|  |  | *A. amoenum* | *A. mono* |
| Flowering | “vegetative to reproductive phase transition of meristem”, “flowering”, “regulation of vegetative phase change” | 6 | 8 |
| Flower development and subsequent processes | “flower development”, “floral whorl structural organization”, “sepal development” | 15 | 9 |
| Regulation of transcriptional processes | “ubiquitin conjugating enzyme activity”, “positive regulation of transcription by RNA polymerase II”, “DNA-binding transcription factor activity” | 15 | 16 |
| Hormonal processes | “response to gibberellin”, “gibberellin biosynthetic process” | 7 | 11 |
| Response to light | “response to red light”, “regulation of photomorphogenesis”, “response to absence of light” | 17 | 6 |
| Circadian rhythm | “Circadian rhythm”, “entrainment of circadian clock by photoperiod”, “circadian regulation of gene expression” | 6 | 1 |
| Vernalization, photoperiodism | “vernalization response”, “photoperiodism, flowering”, “entrainment of circadian clock by photoperiod” | 5 | 6 |
| Others |  | 51 | 44 |
| Total | | 117 | 96 |

(See Table S1 for the list of actual terms.)

Table 4. Significant correlation coefficients (orange for positive and blue for negative) between the expression levels of known flowering-related genes and the expression levels of *FT*s, tree size (H, DBH) and light environment (RPFD, R:FR).

| Flowering pathway | Gene name | *A. amoenum* | | | | | |  | *A. mono* | | | | | |
| --- | --- | --- | --- | --- | --- | --- | --- | --- | --- | --- | --- | --- | --- | --- |
|  |  | *FT1* | *FT2* | H | DBH | RPFD | R:FR |  | *FT1* | *FT2* | H | DBH | RPFD | R:FR |
| Light-dependent pathway (Photoreceptors) | *CRYD^a^* |  | 0.59 | 0.65 | 0.66 | 0.48 | 0.71 |  |  |  |  |  |  |  |
|  | *CRYD* |  | 0.64 | 0.72 | 0.70 | 0.53 | 0.77 |  | 0.50 | 0.44 | 0.71 | 0.62 | 0.46 | 0.67 |
|  | *PHOT1* |  |  |  |  |  |  |  |  | −0.49 | −0.78 | −0.66 | −0.61 | −0.76 |
|  | *PHOT1B* |  |  |  |  |  |  |  |  | −0.50 | −0.82 | −0.69 | −0.60 | −0.79 |
|  | *PHYE* |  |  |  |  |  |  |  |  | −0.46 |  | −0.57 | −0.57 |  |
| Light-dependent pathway (CCT) | *COL4* |  | −0.49 | −0.49 | −0.49 |  | −0.59 |  | −0.45 |  |  |  | −0.47 | −0.50 |
| Light-dependent pathway (other TFs) | *COP1* |  | 0.52 | 0.72 | 0.70 | 0.56 | 0.73 |  | 0.52 |  | 0.81 | 0.66 | 0.58 | 0.80 |
|  | *PHL* |  | 0.47 | 0.64 | 0.57 | 0.63 | 0.64 |  |  |  |  |  |  |  |
|  | *RUP2* |  |  |  |  |  |  |  | 0.55 |  | 0.70 | 0.57 |  | 0.70 |
| Autonomous pathway | *FLC* |  |  |  |  |  |  |  |  | −0.45 | −0.71 | −0.66 | −0.59 | −0.74 |
|  | *FCA ^a^* |  | −0.59 | −0.63 | −0.66 |  | −0.65 |  |  |  |  |  |  |  |
|  | *FCA* |  | −0.53 | −0.48 | −0.46 |  | −0.55 |  |  |  |  |  |  |  |
|  | *FRL3* ***^a^*** |  | −0.63 | −0.65 | −0.66 |  | −0.70 |  | −0.67 | −0.63 | −0.81 | −0.80 | −0.64 | −0.79 |
|  | *FRL4A****^m^*** |  |  |  |  |  |  |  | −0.55 |  | −0.59 |  |  | −0.6 |
|  | *SVP* |  |  |  |  |  |  |  | −0.51 |  | −0.62 | −0.51 |  | −0.63 |
| Age-related pathway | *SPL1* |  |  |  |  |  |  |  |  | 0.51 | 0.61 | 0.61 | 0.49 | 0.51 |
|  | *SPL3* |  | 0.6 | 0.61 | 0.58 |  | 0.68 |  |  |  |  |  |  |  |
|  | *SPL4* ***^m^*** |  | 0.6 | 0.61 | 0.58 |  | 0.68 |  |  | 0.47 |  |  |  | 0.52 |
|  | *SPL5* ***^a^*** |  | 0.45 | 0.54 | 0.56 | 0.53 | 0.58 |  |  |  |  |  |  |  |
|  | *SPL12* |  | −0.44 | −0.53 | −0.56 |  | −0.52 |  |  |  |  |  |  |  |
| Sugar-mediated pathway | *CTR1* |  |  |  |  |  |  |  | −0.50 | −0.54 | −0.64 | −0.69 | −0.58 | −0.68 |
|  | *CTR1* |  | 0.55 | 0.67 | 0.63 | 0.52 | 0.7 |  |  |  |  |  |  |  |
|  | *EDR1* |  |  |  |  |  |  |  | −0.52 |  | −0.44 |  |  | −0.5 |

Relevant protein names: *COP1*, Constitutive photomorphogenesis protein 1; *CRYD*, Cryptochrome DASH; *CTR1*, Protein CONSTITUTIVE TRIPLE RESPONSE 1; *EDR1*, Protein ENHANCED DISEASE RESISTANCE 1; *FCA*, Flowering time control protein FCA; *FRL3/4A*, FRIGIDA-like protein 3/4a; *PHL*, Protein PHYTOCHROME-DEPENDENT LATE FLOWERING; *PHOT1/1B*, Phototropin-1/1B; *PHYE*, Phytochrome E; *SPL*s, Squamosa promoter-binding-like proteins; *SVP*, Protein SHORT-VEGETATIVE PHASE; *RUP2*, Protein REPRESSOR OF UV-B PHOTOMORPHOGENESIS 2; *VRN1*, Protein VERNALIZATION 1.

Multiple transcripts may be annotated as mRNAs for the same protein. Gray font: Marginally significant (p < 0.015). **a** and **m** as superscript: Genes that demonstrated significant response to light manipulation in *A. amoenum* (a) and *A. mono* (m) samples. Vacant cells: No significant correlation.

Table 5. Flowering-related genes whose expression levels significantly changed as trees grew and were significantly up- (orange) or down- regulated (blue) under high RPFD or R:FR conditions in the light manipulation experiment.

*A. amoenum*

| Gene name (similarity %) | β_RPFD_ | *P*(*t*) | β_R:FR_ | *P*(*t*) | TE |
| --- | --- | --- | --- | --- | --- |
| *ATH17* (63.3%) | −0.01 | 0.003 | −0.46 | 0.065 | * |
| *BRR2A* (58.06%) | −0.01 | 0.036 | 0.45 | 0.845 | * |
| *CERBERUS* (63.12%) | 0.01 | 0.915 | −2.08 | 0.022 | * |
| ***CRYD* (64.64%)** | 0.02 | 0.050 | −0.35 | 0.714 |  |
| ***FCA* (50.23%)** | 0.01 | 0.126 | −0.73 | 0.037 |  |
| ***FRL3* (55.98%)** | 0.00 | 0.023 | 0.13 | 0.757 | * |
| *FULL* (67.09%) | 0.01 | 0.999 | −0.94 | 0.002 | * |
| *LUG* (81.75%) | 0.00 | 0.040 | 0.05 | 0.617 | * |
| *MYBS2* (70.48%) | −0.01 | 0.001 | 0.44 | 0.941 | * |
| ***SPL5* (73.9%)** | 0.01 | 0.904 | −0.92 | 0.045 | * |
| *UBC2* (72.66%) | 0.00 | 0.035 | −0.02 | 0.456 | * |
| *UBC2* (83.3%) | −0.01 | 0.003 | 0.53 | 0.945 | * |

| *A.mono* | | | | | |
| --- | --- | --- | --- | --- | --- |
| Gene name (similarity %) | β_RPFD_ | *P*(*t*) | β_R:FR_ | *P*(*t*) | TE |
| *ARR12* (65.27%) | −0.01 | 0.017 | 0.57 | 0.036 |  |
| *ATHB7* (73.6%) | −0.04 | 0.016 | 1.28 | 0.827 | * |
| *CIA2* (71.62%) | 0.01 | 0.990 | −1.20 | 0.003 | * |
| *CPC* (69.64%) | −0.03 | 0.012 | 1.89 | 0.956 | * |
| ***FRL4A* (61.94%)** | −0.01 | 0.036 | 0.60 | 0.981 | * |
| *GAF1* (78.67%) | −0.01 | 0.009 | 0.16 | 0.642 | * |
| *HAT5* (73.69%) | −0.01 | 0.002 | 0.32 | 0.857 | * |
| *MYB60* (76.77%) | −0.01 | 0.000 | 0.24 | 0.828 | * |
| *NUP98A* (67.99%) | 0.00 | 0.006 | 0.24 | 0.046 |  |
| ***SPL4* (74.24%)** | 0.01 | 0.992 | −0.60 | 0.040 | * |
| *y1* (80.53%) | 0.05 | 0.110 | −5.58 | 0.046 |  |

β_RPFD_ and β_R:FR_: Linear regression coefficients of gene expression levels and RPFD or R:FR.

TE: The effect of the individual tree sampled was not negligible for genes with asterisks.

**Bold** font: Flowering-related TFs that are also listed in Table 4. Relevant protein names for these genes are in the footnote of Table 4.

**Figure Legends**

**Fig. 1** Numbers of DEGs that were correlated (*p* *<* 0.001) with each of the four parameters in *A. amoenum* (top) and *A. mono* (bottom).

Alt text: Three graphical representations of the number of differentially expressed genes (DEG) that are correlated with each of *AaFT2*, *AmFT1*, and *AmFT2*. In each graph, about half of all DEGs are correlated with both tree size and light environments.

**Fig. 2** Heatmap of normalized expression levels of DEGs in *A. amoenum* trees, along with the tree size (H, DBH) and light environment (RPFD and R:FR) in the bargraphs below.

Clusters show genes (rows) and trees (columns) with similar expression patterns. Arrows enhance FT. Broken lines separate major clusters and subclusters.

Row names: probable gene name (i.e., ortholog name, with % similarity).

*: These transcripts were annotated as *COP1* but lack *COP1*-characteristic alignments.

Alt text: Graphical representation of DEG expression patterns in *Acer amoenum* trees. A clear gap is present in expression levels of most DEGs between saplings and large trees.

**Fig. 3** Heatmap of normalized expression levels of DEGs in *A. mono*, along with the tree size (H, DBH) and light environment (RPFD and R;FR) in the bargraphs below.

Clusters show genes (rows) and trees (columns) with similar expression patterns. Arrows enhance FT. Broken lines separate major clusters and subclusters.

Row names: probable gene name (i.e., ortholog name, with % similarity).

Alt text: Graphical representation of DEG expression patterns in *Acer mono* trees. For this species, the expression pattern differs not only between saplings and adults, but also among large trees, depending on tree height and red-to-far-red ratio.

**Fig. 4** Height- and R:FR-dependent variation in the expression levels of *FLOWEING LOCUS T* (*FT1*, gray circles and dotted line; *FT2*, white circles and solid line) and *FLOWEING LOCUS C* (*FLC*) in *A. amoenum* (top) and *A. mono* (bottom).

Linear regression lines of best fit were plotted if the correlation was significant (*t*-test, *p* < 0.01).

Alt text: Graphs showing the comparison between *FT* or *FLC* expression and tree height or red-far-red ratios for each species. For *Acer mono*, *FT*s increased and *FLC* decreased in taller and brighter trees. For *Acer amoenum*, only *FT2* increased as the trees grew.

**Fig. 5** Significant correlations between the expression levels of *FLOWEING LOCUS T* (*FT1/2*) and autonomous-pathway genes, as well as the light-dependent pathway gene *COP1* in *A. amoenum* (top) and *A. mono* (bottom). The autonomous pathway genes code FRIGIDA-like protein 3/4a (*FRL3*/*4A*) and Flowering time controlling protein FCA (*FCA*).

Linear regression lines were drawn with dotted and solid lines for *p* < 0.01 and *p* < 0.001 cases, respectively (*t*-test).

Alt text: Graphs showing the correlated expression changes between *FT*, *COP1*, *FRL3*, *FCA*, and *FRL4A*. *COP1* level showed positive correlation with *FT* level; others showed negative correlations.

**Fig. 6** Significant correlations between the expression levels of *FLOWEING LOCUS T* (*FT1/2*) and age-dependent pathway genes that code protein squamosa promoter-binding protein-like (*SPLs*) in *A. amoenum* (top) and *A. mono* (bottom).

Linear regression lines were drawn with dotted and solid lines for *p*<0.01 and *p*<0.001 cases, respectively (*t*-test).

Alt text: Graphs showing the positive correlation in expression levels between *FT* and *SPL*s in both species.
