# Supplementary material for: Genetic mechanisms for size- and light-dependent reproductive maturation in two maple species, Acer amoenum var. amoenum and Acer mono var. glabrum
Source: Ann Bot. 2026 Apr 24;138(2):596–610. doi: 10.1093/aob/mcag111 (PMC13454698; doi:10.1093/aob/mcag111)
Supplement: Supplementary file 1 [file mcag111_supplementary_data.zip › AppendixS1.pdf]

Appendix S1: Details on the *FLOWERING LOCUS T* detection process from the RNA-seq data

As described in the MATERIALS AND METHODS section, we mapped RNA-seq data of *Acer amoenum* (*Aa*) and *Acer mono* (*Am*) using the reference genomes of their close relatives, *A. pseudosieboldianum* and *A. truncatum*, respectively. We then searched for annotated transcripts of the *FLOWERING LOCUS T* (*FT*) gene within the contigs of both species. One *FT* sequence with high homology was confirmed in *Am* contigs, whereas two *FT* sequences with high homology were identified in *Aa* contigs. To investigate this discrepancy, we closely examined the *A. truncatum* genome sequence used as the reference genome for *Am*. We concluded that the transcript annotated as *FT* (Atru.chr12.1642_Atru.chr12.1636) most likely represents the fusion of two original *FT* genes. To support this conclusion, we performed de novo assembly using floral organ RNA-seq data registered by the authors who constructed this genome (SRR10097463; Ma et al., 2020). This yielded two *FT* sequences, provisionally named *Atru-FT1* and *Atru-FT2*. Sequence comparison with the exon sequences of Atru.chr12.1642_Atru.chr12.1636 revealed that the first half is similar to the *Atru-FT1* sequence and the second half is comparable to the *Atru-FT2* sequence. Therefore, Atru.chr12.1642_Atru.chr12.1636 is considered a chimera originating from the fusion of the *Atru-FT1* sequence and the *Atru-FT2* sequence. This may have occurred due to an error during genome sequence construction. However, identifying the chromosomal positions of the two *FT* sequences based on the current *A. truncatum* genome sequence information has been challenging.

The two *FT* sequences of *A. truncatum* (*Atru-FT1* and *Atru-FT2*) constructed via the aforementioned de novo assembly demonstrated sequence identity rates of 98.9% and 99.4%, respectively, with the two *FT* sequences (provisionally named *Apse-FT1* and *Apse-FT2*) identified from the *A. pseudosieboldianum* genome sequence (GWHPBECT008371 and GWHPBECT008372). *Am* data mapped using the *A. pseudosieboldianum* reference genome successfully annotated two transcripts as Apse-FT1 and Apse-FT2 with high similarity.

Therefore, the *FT* expression levels in both maple species (*Aa* and *Am*) were quantified using count data from two *FT*s identified from *Aa* and *Am* transcripts, with the *A. pseudosieboldianum* genome sequence as a reference.
