# Supplementary material for: Genetic mechanisms for size- and light-dependent reproductive maturation in two maple species, Acer amoenum var. amoenum and Acer mono var. glabrum
Source: Ann Bot. 2026 Apr 24;138(2):596–610. doi: 10.1093/aob/mcag111 (PMC13454698; doi:10.1093/aob/mcag111)
Supplement: Supplementary file 1 [file mcag111_supplementary_data.zip › FigS1.pdf]

Fig. S1

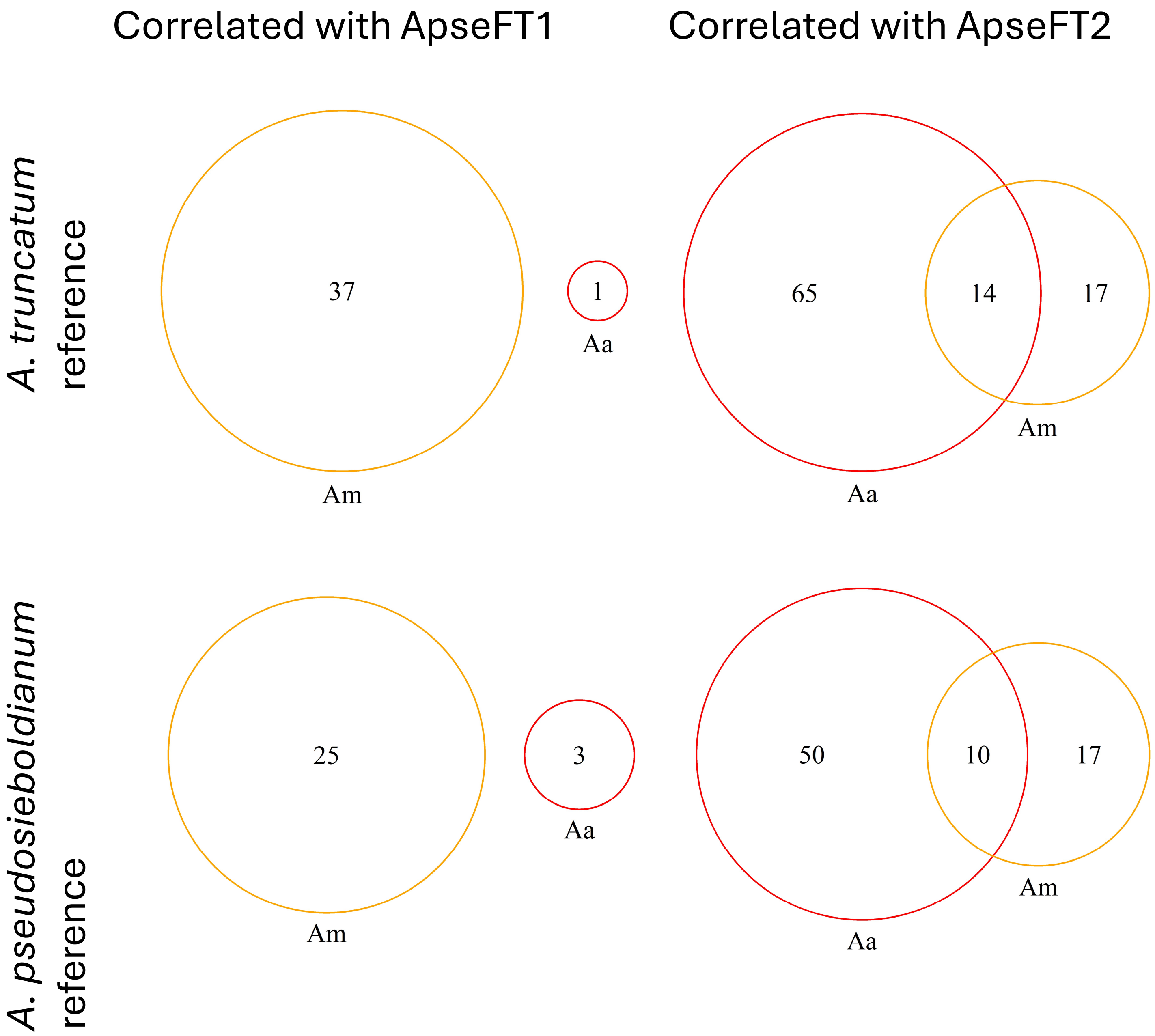
Correlated with ApseFT1
Correlated with ApseFT2
A. truncatum reference
37
Am
1
Aa
65
14
17
Aa
Am
A. pseudosieboldianum reference
25
Am
3
Aa
50
10
17
Aa
Am
