# Supplementary material for: Genetic mechanisms for size- and light-dependent reproductive maturation in two maple species, Acer amoenum var. amoenum and Acer mono var. glabrum
Source: Ann Bot. 2026 Apr 24;138(2):596–610. doi: 10.1093/aob/mcag111 (PMC13454698; doi:10.1093/aob/mcag111)
Supplement: Supplementary file 1 [file mcag111_supplementary_data.zip › TableS1.pdf]

Supplementary Table S1. Summary of the data filtering process.

Numbers of transcripts	Reference genome	
	Acer truncatum	*A. pseudo-sieboldianum*
i. All transcripts in RNA-seq data	28437	29679
ii. Transcripts dissimilar to all Swiss–Prot genes	7574	8453
iii. Transcripts annotated to Swiss–Prot genes whose GONames don't include "flower" nor "vegetative to reproductive"	19240	19594
iv. Remaining transcripts (i − ii − iii)	1623	1632
v-i. Transcripts expressed in five or more *A. amoenum* samples	1400	**1437**
v-ii. Transcripts expressed in five or more *A. mono* samples	**1395**	1413

Bold: The numbers of transcripts on which the correlation tests were finally conducted.
