# Supplementary material for: Genetic mechanisms for size- and light-dependent reproductive maturation in two maple species, Acer amoenum var. amoenum and Acer mono var. glabrum
Source: Ann Bot. 2026 Apr 24;138(2):596–610. doi: 10.1093/aob/mcag111 (PMC13454698; doi:10.1093/aob/mcag111)
Supplement: Supplementary file 1 [file mcag111_supplementary_data.zip › TableS3.pdf]

Supplementary Table S3. Correlation test results ($p < 0.01$) for transcripts annotated as major transcription factors of known flowering pathways.

1. Tested TFs in *Acer amoenum* RNA-seq data (reference genome: *A. pseudosieboldianum*)

Search key for transcription factor	Abbreviation	DEG test result (Pass/Fail)
Flowering locus C (according to Acer-limited BLAST search)	*FLC*	Fail
Protein MOTHER of FT and TFL1	*MFT*	Fail
**Light-dependent pathway genes:**		
Cyclic dof factor 2	*CDF2*	Fail
Cyclic dof factor 3	*CDF3*	Fail
Zinc finger protein HD1 (CONSTANS-like protein of *Oryza*)	*CO*	Fail
CONSTANS-LIKE 2	*COL2*	Fail
CONSTANS-LIKE 3	*COL3*	Fail
CONSTANS-LIKE 4	*COL4*	Pass
CONSTANS-LIKE 5	*COL5*	Fail
CONSTANS-LIKE 6	*COL6*	Fail
CONSTANS-LIKE 7	*COL7*	Fail
CONSTANS-LIKE 9	*COL9*	Fail
CONSTANS-LIKE 12	*COL12*	Fail
CONSTANS-LIKE 13	*COL13*	Fail
CONSTANS-LIKE 14	*COL14*	Fail
E3 ubiquitin transferase COP1*	*COP1*	Pass
Cryptochrome DASH	*CRYD*	Pass
Cullin-4	*CUL4*	Fail
ELF3-like	*ELF3*	Fail
E3 ubiquitin-protein ligase HOS1	*HOS1*	Fail
Protein PHYTOCHROME-DEPENDENT LATE-FLOWERING	*PHL*	Pass
Light-sensor Protein kinase	*PHY*	Fail
Phytochrome type A	*PHYA*	Fail
Phytochrome A	*PHYA*	Fail
Phytochrome B	*PHYB*	Fail
Phytochrome C	*PHYC*	Fail

Phytochrome E	*PHYE*	Fail
SPA1-Related 2	*SPA2*	Fail
SPA1-Related 3	*SPA3*	Fail
VOZ1	*VOZ1*	Fail
VOZ2	*VOZ2*	Fail
ZEITLUPE	*ZTL*	Fail
**Autonomous pathway genes:**		
Flowering time control protein FCA	*FCA*	Pass
Flowering locus K homology domain	*FLKH*	Fail
Flowering time control protein FPA	*FPA*	Fail
Protein FRIGIDA	*FRI*	Fail
FRIGIDA-like protein 4a	*FRI4A*	Fail
FRIGIDA-like protein 4b	*FRI4B*	Fail
FRIGIDA-like protein 1	*FRL1*	Fail
FRIGIDA-like protein 3	*FRL3*	Pass
Flowering time control protein FY	*FY*	Fail
Homeobox protein LUMINIDEPENDENS	*LD*	Fail
Protein FLOWERING LOCUS D-LIKE	*LDL1*	Fail
**Age-dependent pathway genes:**		
Squamosa promoter-binding-like protein 1	*SPL1*	Fail
Squamosa promoter-binding-like protein 12	*SPL12*	Pass
Squamosa promoter-binding-like protein 15	*SPL17*	Fail
Squamosa promoter-binding-like protein 3	*SPL3*	Pass
Squamosa promoter-binding-like protein 4	*SPL4*	Pass
Squamosa promoter-binding-like protein 5	*SPL5*	Pass
Squamosa promoter-binding-like protein 6	*SPL6*	Fail
Squamosa promoter-binding-like protein 7	*SPL7*	Fail
Squamosa promoter-binding-like protein 8	*SPL8*	Fail
**Gibberellin (GA) pathway genes:**		
Agamous-like MADS-box protein AGL15	*AGL15*	Fail
Protein CONSTITUTIVE TRIPLE RESPONSE1	*CTR1*	Pass
DELLA	*DELLA*	Fail
GRAS family protein 9	*DELLA*	Fail
Protein HEADING DATE 16	*HD16*	Pass
TEM1	*TEM1*	Fail

WRKY75	*WRKY75*	Pass
**Sugar-mediated pathway genes:**		
Agamous-like MADS-box protein AGL42	*AGL42*	Fail
Agamous-like MADS-box protein AGL61	*DIANA*	Fail
MAPKK kinase EDR1	*EDR1*	Fail
Protein SUGAR INSENSITIVE 8	*SIS8*	Fail
**Vernalization-related genes:**		
Protein SHORT VEGETATIVE PHASE	*SVP*	Fail
VRN1	*VRN1*	Fail

*Multiple transcripts were annotated as *COP1*; however, only two of them were considered real *COP1* based on its COP1-specific structure (Zinc-finger + coiled-coil + WD40 repeat).

2. Tested TFs in *Acer mono* RNA-seq data (reference genome: *A. pseudosieboldianum*)

Search key for transcription factor	Abbreviation	DEG test result (Pass/Fail)
Flowering locus C (according to Acer-limited BLAST search)	*FLC*	Pass
Protein MOTHER of FT and TFL1	*MFT*	Fail
**Light-dependent pathway genes:**		
Cyclic dof factor 3	*CDF3*	Fail
Cyclic dof factor 2	*CDF2*	Fail
Zinc finger protein HD1	*CO*	Fail
CONSTANS-LIKE CO3	*CO3*	Fail
CONSTANS-LIKE 2	*COL2*	Fail
CONSTANS-LIKE 3	*COL3*	Fail
CONSTANS-LIKE 4	*COL4*	Pass
CONSTANS-LIKE 5	*COL5*	Fail
CONSTANS-LIKE 6	*COL6*	Fail
CONSTANS-LIKE 7	*COL7*	Fail
CONSTANS-LIKE 9	*COL9*	Fail
CONSTANS-LIKE 12	*COL12*	Fail
CONSTANS-LIKE 13	*COL13*	Fail
CONSTANS-LIKE 14	*COL14*	Fail
E3 ubiquitin-protein ligase COP1*	*COP1*	Pass
Cryptochrome-1	*CRY1*	Fail

Cryptochrome-2	*CRY2*	Fail
Cryptochrome DASH	*CRYD*	Pass
Cullin-4	*CUL4*	Fail
ELF3-like	*ELF3*	Fail
Protein HIGH EXPRESSION OF OSMOTICALLY RESPONSIVE GENE 1	*HOS1*	Fail
Protein PHYTOCHROME-DEPENDENT LATE-FLOWERING	*PHL*	Fail
Light-sensor Protein kinase	*PHY*	Fail
Phototropin-1	*PHOT1*	Pass
Phototropin-1B	*PHOT1B*	Pass
Phototropin-2	*PHOT2*	Fail
Phytochrome	*PHY*	Fail
Phytochrome type A	*PHYA*	Fail
Phytochrome A	*PHYA*	Fail
Phytochrome B	*PHYB*	Fail
Phytochrome C	*PHYC*	Fail
Phytochrome E	*PHYE*	Pass
WD repeat-containing protein RUP2	*RUP2*	Pass
SPA1-Related 2	*SPA2*	Fail
SPA1-Related 3	*SPA3*	Fail
AT-hook motif nuclear-localized protein 16	*TEK*	Fail
VOZ1	*VOZ1*	Fail
VOZ2	*VOZ2*	Fail
ZEITLUPE	*ZTL*	Fail
**Autonomous pathway genes:**		
Flowering time control protein FCA	*FCA*	Fail
Protein FLOWERING LOCUS D	*FLD*	Fail
Flowering locus KH domain	*FLKH*	Fail
Flowering time control protein FPA	*FPA*	Fail
FRIGIDA-like protein 1	*FRIL1*	Fail
FRIGIDA-like protein 3	*FRL3*	Pass
FRIGIDA-like protein 4a	*FRI4A*	Pass
FRIGIDA-like protein 4b	*FRI4B*	Fail
Protein FRIGIDA	*FRI*	Fail

Flowering time control protein FY	*FY*	Fail
Homeobox protein LUMINIDEPENDENS	*LD*	Fail
**Age-dependent pathway genes:**		
Squamosa promoter-binding-like protein 1	*SPL1*	Pass
Squamosa promoter-binding-like protein 4	*SPL4*	Pass
Squamosa promoter-binding-like protein 5	*SPL5*	Fail
Squamosa promoter-binding-like protein 7	*SPL7*	Fail
Squamosa promoter-binding-like protein 8	*SPL8*	Fail
Squamosa promoter-binding-like protein 12	*SPL12*	Fail
Squamosa promoter-binding-like protein 17	*SPL17*	Fail
**Gibberellin pathway genes:**		
DELLA	*DELLA*	Fail
GRAS family protein 9	*DELLA*	Fail
Protein HEADING DATE 16	*HD16*	Pass
TEM1	*TEM1*	Fail
WRKY75	*WRKY75*	Pass
**Sugar-mediated pathway genes:**		
AGL42	*AGL42*	Fail
Protein CONSTITUTIVE TRIPLE RESPONSE1	*CTR1*	Pass
MAPKK kinase EDR1	*EDR1*	Pass
**Vernalization related genes:**		
Protein SHORT VEGETATIVE PHASE	*SVP*	Pass
VRN1	*VRN1*	Fail

*Multiple transcripts were annotated as *COP1*; however, only one of them was considered real *COP1* based on its COP1-specific structure (Zinc-finger + coiled-coil + WD40 repeat).
